# Fetal programming in ruminant animals: understanding skeletal muscle development to improve meat quality

**DOI:** 10.1093/af/vfab076

**Published:** 2021-12-17


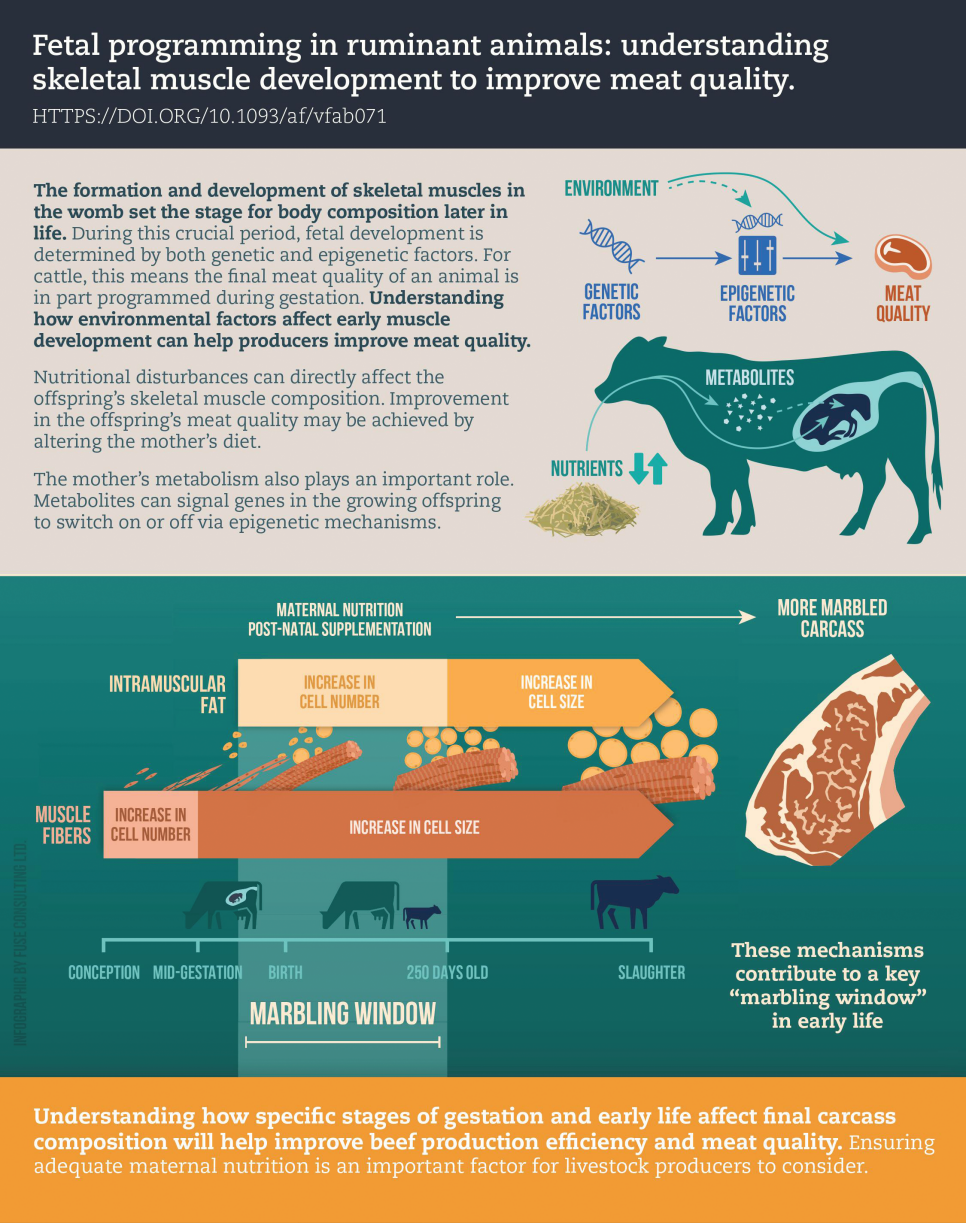


This issue focuses on how nutrition and environment interacts with the epigenome and may influence phenotypic expression. The ultimate consequences on animal production and animal health are examined for their potential application to animal agriculture (Schenkel, 2021).
